# Correction to: Activation of LncRNA TINCR by H3K27 acetylation promotes Trastuzumab resistance and epithelial-mesenchymal transition by targeting MicroRNA-125b in breast Cancer

**DOI:** 10.1186/s12943-021-01385-9

**Published:** 2021-06-11

**Authors:** Huaying Dong, Jianguo Hu, Kejian Zou, Mulin Ye, Yuanwen Chen, Chengyi Wu, Xin Chen, Mingli Han

**Affiliations:** 1 Department of General Surgery, Hainan General Hospital, Hainan Medical University, No.19 Xiu Hua Road, Xiuying District, Haikou City, 570311 Hainan Province China; 2 Department of Obstetrics and Gynecology, The Second Affiliated Hospital, Chongqing Medical University, Chongqing, 400010 China; 3 Department of General Surgery, Chongqing Renji Hospital, University of Chinese Academy of Science, Chongqing, 400062 China; 4 Department of General Surgery, The Frist Affiliated Hospital, Chongqing Medical University, Chongqing, 400016 China; 5 Department of Breast Surgery, The First Affiliated Hospital of Zhengzhou University, Zhengzhou, 450052 China

**Correction to: Mol Cancer 18, 3 (2019)**

**https://doi.org/10.1186/s12943-018-0931-9**

Following the publication of the original paper [1], the authors realized that there are errors in Fig. 3d and Fig. 6e, which caused duplications. The errors occurred during the preparation of figures and went unnoticed during the review process and during preparation of final publication. The revised Figures are shown below. This correction does not alter any of the findings or conclusions of the study. The authors regret this error.

The specific changes of figures are listed as follows:

1. Figure 3d (Replacement for *48h of SKBR-3TR cells*),

2. Figure 6e (Replacement for *0h (sh-TINCR#1+p-Snail-1) of SKBR-3TR cells*)

(insert figures here)

**Fig. 3:**
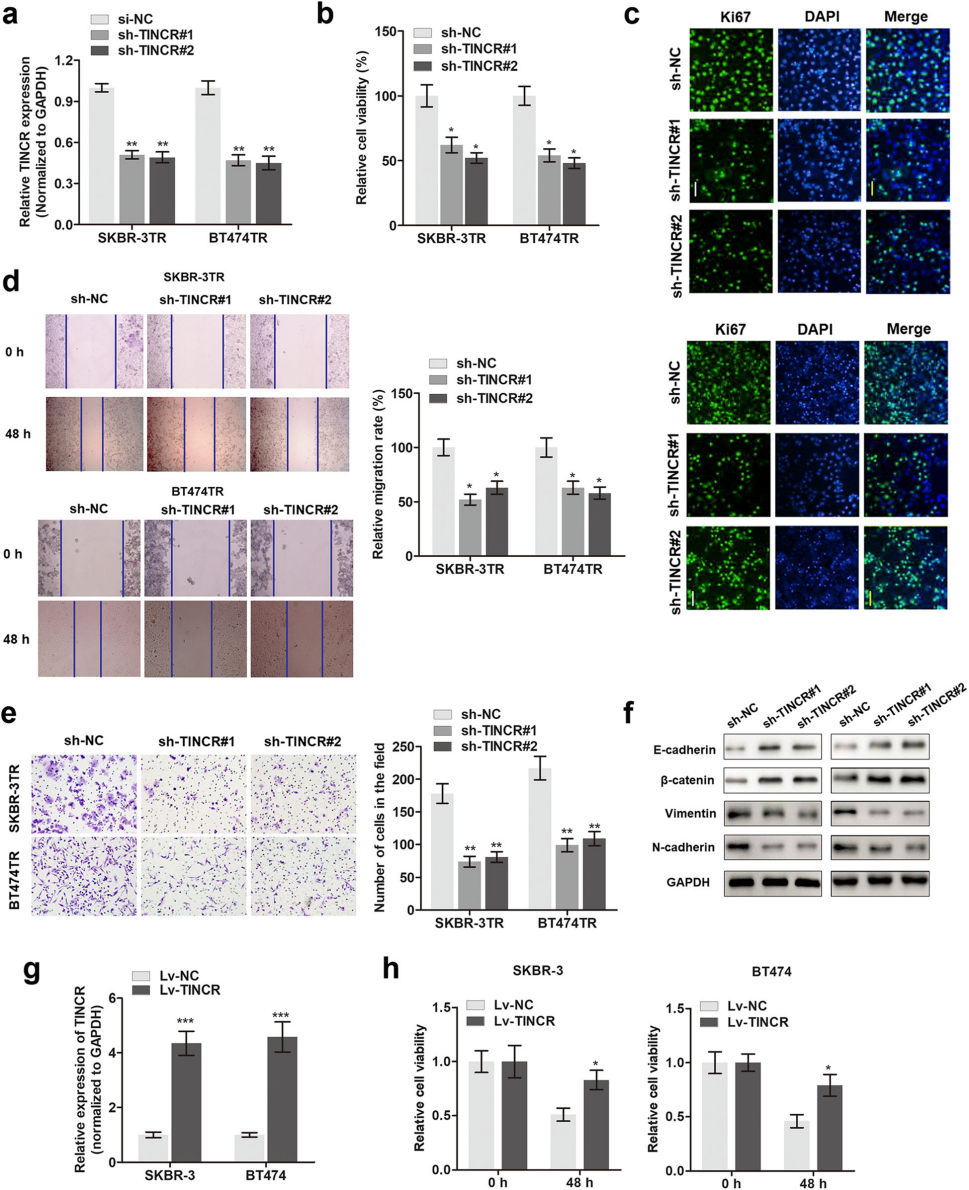
Knockdown of TINCR abrogated trastuzumab resistance of breast cancer cells. **a** qPCR determination of the silencing effect of TINCR after infection with sh-TINCR#1 and sh-TINCR#2. ^**^P < 0.01 compared to sh-NC group. **b** Cell viability was measured by MTT assay in cells silenced with TINCR. ^*^ *P* < 0.05 compared to sh-NC group. **c** In site Ki-67 expression was detected by performing immunofluorescence assay (Images were magnified at 20×). **d** Migration ability was assessed by using Wound-healing assay in cells silenced with TINCR. ^*^ *P* < 0.05 compared to sh-NC group. **e** Invasion ability was assessed by using Matrigel transwell assay. ^**^P < 0.01 compared to sh-NC group. **f** The expression levels of Ecadherin, β-catenin, vimentin and N-cadherin were determined by Western blot assay. **g** TINCR expression was assessed via q-PCR in cells infected with Lv-TINCR. ^***^P < 0.001 compared to Lv-NC group. **h** Cell viability was determined via MTT assay in breast cancer cells infected with Lv-TINCR. ^*^ *P* < 0.05 compared to Lv-NC group

**Fig. 6:**
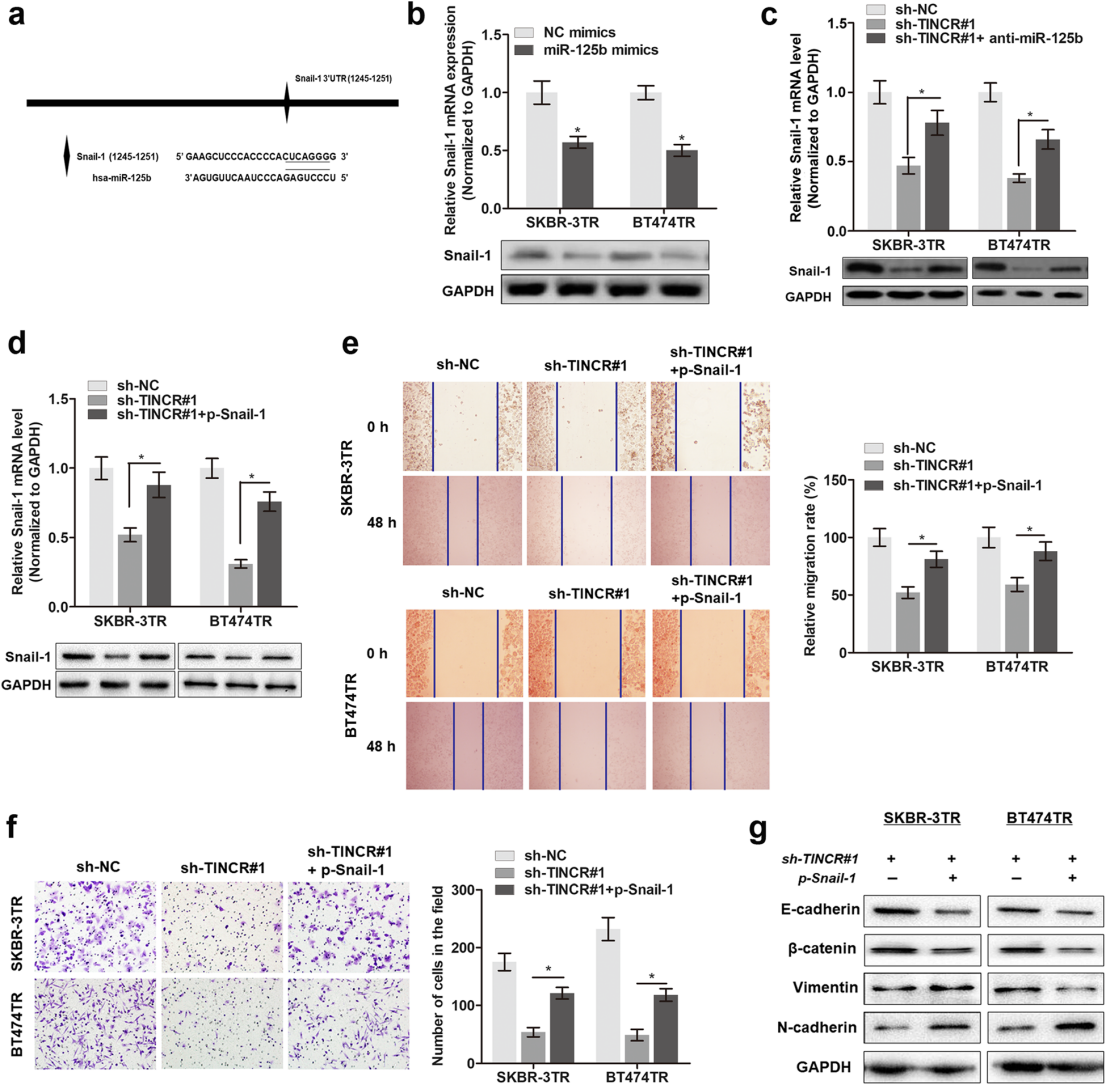
TINCR regulates trastuzumab-induced EMT by targeting Snail-1. **a** The putative sequences of binding site between miR-125b and TINCR. **b** The mRNA and protein levels of Snail-1 was assessed by qPCR and Western blot in cells overexpressed with miR-125b. ^*^ *P* < 0.05 compared to NC mimics. **c** anti-miR-125b rescued the shTINCR-induced inhibition of Snail-1 expression as evidenced by qPCR and Western blot assay, ^*^ *P* < 0.05. **d** Snail-1 was overexpressed by transfection of p-Snail-1, ^*^ *P* < 0.05. **e** Cell migration ability was assessed by Wound-healing assay in cells silenced with TINCR or (and) overexpressed with Snail-1, ^*^ *P* < 0.05. **f** Cell invasion ability was assessed by Transwell assay in cells silenced with TINCR or (and) overexpressed with Snail-1, ^*^ *P* < 0.05. **g** The expression levels of E-cadherin, β-catenin, vimentin and N-cadherin were determined by Western blot assay
